# Peripapillary retinal nerve fiber layer and macular ganglion cell complex thickness in patients with chronic phase of nonarteritic anterior ischemic optic neuropathy

**Published:** 2018

**Authors:** Muhammed Şahin, Alparslan Şahin, Fatih Mehmet Türkcü, Hamza Aslanhan, Harun Yüksel

**Affiliations:** * Department of Ophthalmology, Dicle University, School of Medicine, Diyarbakir, Turkey; ** Department of Ophthalmology, Batman Private Hospital, Batman, Turkey; *** Department of Ophthalmology, Batman Zilan Hospital, Batman, Turkey; **** Department of Family Medicine, Medical Faculty of Dicle University, Diyarbakir, Turkey; ***** Frelance Physician, Diyarbakır, Turkey

**Keywords:** central macular thickness, macular ganglion cell complex, nonarteritic anterior ischemic optic neuropathy, peripapillary retinal nerve fiber layer

## Abstract

**Objective:** We aimed to investigate the peripapillary retinal nerve fiber layer (pRNFL) and macular ganglion cell complex (mGCC) thickness in patients with chronic phase of nonarteritic anterior ischemic optic neuropathy (crNAION) analyzed by spectral domain optical coherence tomography (SD-OCT).

**Methods:** Patients with crNAION, and healthy control subjects were enrolled in the study. All participants underwent SD-OCT for measurement of pRNFL, mGCC, and central macular thickness (CMT). The measurements of the eyes of the patients with crNAION were compared with those of the control subjects and unaffected fellow eyes.

**Results:** A total of 25 patients with crNAION were eligible for the study. The control group consisted of 50 healthy subjects. The pRNFL and mGCC thickness in eyes with crNAION were found to be significantly thinner in all quadrants when compared to those of healthy control subjects and unaffected fellow eyes. The CMT of the eyes with crNAION was similar to that of the healthy control subjects.

**Conclusions:** We demonstrated that mGCC and pRNFL thickness measurement by SD-OCT are capable of detecting axonal damage in eyes with crNAION. Furthermore, this study used SD-OCT and found that mGCC and pRNFL had the ability to detect GC loss in the eyes of the patients with crNAION.

## Introduction

Nonarteritic anterior ischemic optic neuropathy (NAION) is a common vascular disease in elderly patients presenting with unilateral, painless, sudden vision loss with optic disc edema [**1**–**3**]. It is the second most common optic neuropathy after glaucoma, with a recently estimated incidence of 82 cases per 100,000, of which the population is over 67 years old [**4**].

Nonarteritic anterior ischemic optic neuropathy is caused by acute ischemic damage of the anterior region of the optic nerve due to reduced perfusion of short posterior ciliary arteries and afterwards it leads to apoptosis of retinal ganglion cells (RGCs) and optic nerve atrophy [**5**]. The RGCs comprise three layers in the retina: the mRNFL (macular GC axons), the GC layer (GC bodies), and the IPL (GC dendrites). All three layers are defined as the GCC [**6**]. 

In an experimental AION model, Lee et al. [**7**] demonstrated that RGC axons showed severe degenerative changes within 1 week after the acute ischemic event. The pRNFL thickness was significantly increased by disc swelling that may mask retinal ganglion cell loss in the acute stage of disease. The GCIPL thickness is less likely to be affected by optic disc edema in the acute phase of NAION compared to RNFL. Moreover, GCIPL might be useful for the detection of the axonal loss over time with OCT.

In the chronic phase of NAION (crNAION), degeneration of RGCs is usually observed after the acute phase. The pallor of the optic disc is a subjective method to evaluate the loss of ganglion cells and their axons. Histological studies of the eyes with NAION have demonstrated significant damage in the optic nerve axons [**8**]. One might be able to indirectly document the loss of RGC by measuring the macular ganglion cell complex (mGCC) and the peri-papillary retinal nerve fiber layer (pRNFL), since the aforementioned provide quantitative outcomes [**6**].

Spectral domain optical coherence tomography (SD-OCT) imaging is a useful device used in neuro-ophthalmological diseases [**9**]. The majority of the imaging and SD-OCT studies conducted on NAION aimed to analyze the optic disc and peri-papillary retina, however few studies have focused on macular structure [**10**,**11**].

The measurement of the macular structure may provide more useful information in neuro-ophthalmological diseases, such as glaucoma, and NAION, since the axons of the RGC are directly involved in the optic nerve pathology and their preservation is important to the prevention of the vision loss. Previously, it has been reported that macular thickness is correlated with visual field (VF) loss [**12**], the thinning of the GC and inner plexiform layer (IPL) and the pRNFL [**6**]. Moreover, a correlation between GC+IPL thickness and the contralateral hemispheric loss of VF was reported in NAION [**13**].

Quantification of pRNFL and mGCC thickness changes is likely to enhance our knowledge of the pathophysiology and natural history of NAION. We aimed to investigate both pRNFL and mGCC in patients with crNAION that was analyzed by SD-OCT.

## Method

The study protocol was approved by the local Ethics Committee and was conducted in accordance with the Declaration of Helsinki. The patients who were diagnosed as chronic NAION between January 2013 and May 2015 were reviewed retrospectively. Each subject underwent a complete ocular examination including the measurement of Snellen visual acuity (VA), anterior segment examination, Goldmann applanation tonometry, and fundoscopic examination. The SD-OCT images of the participants were obtained. The demographic data including age, gender, ocular pathology, and presence of systemic disease were recorded. The crNAION was defined as having at least six months of the NAION symptoms duration from onset. The diagnosis of crNAION was made upon ocular examination, including a detailed history, VA, VF, fundus examination, and VFD consistent with NAION.

Patients with neurological disease, amblyopia, history of papilledema or glaucoma, those with diabetic retinopathy or other retinal and macular disease and/ or with a history of any ocular surgery or trauma and/ or chronic ocular corticosteroid use, those with ocular hypertension, spherical refraction higher than ±3.00 diopters and astigmatism higher than ±2.00 diopters were excluded. Age and gender matched subjects who admitted outpatient clinic of ophthalmology for spectacle correction (within +/ -2 diopters) comprised the control group.

**SD-OCT Scanning**

**Fig. 1:**
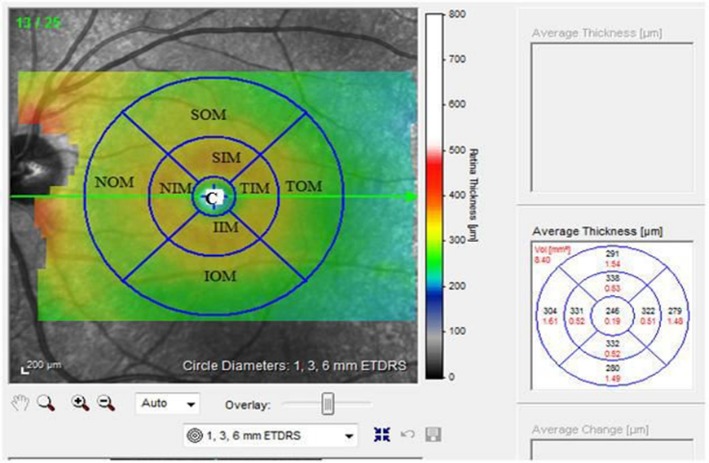
Representative OCT image of the nine ETDRS subfield C = central, SIM = superior inner quadrant in the macula, NIM = nasal inner quadrant, IIM = inferior inner quadrant, TIM = temporal inner quadrant, SOM = superior outer quadrant in the macula, NOM = nasal outer quadrant, IOM = inferior outer quadrant, TOM = temporal outer quadrant

We obtained SD-OCT (Spectralis; Heidelberg Engineering, Heidelberg, Germany) measurements for each eye. Furthermore, all the subjects underwent the same acquisition protocol, including a peri-papillary scan with 3.5 mm diameter circle scans with automatic real time (ART) of 90 and a macular raster scan centered on the fovea (20º×20º matrix and 25 horizontal sections with 240 µm of separation and a mean ART of 45). Retinal layer segmentation was performed automatically using the Spectralis OCT. Then, we quantified the thickness of the following layers: (1) pRNFL (nasal, superonasal, superotemporal, temporal, inferotemporal, and inferonasal, as well as global RNFL thickness), (2) mGCC; GC layer + IPL + macular RNFL (mRNFL), and central macular thickness (CMT). For the study, the measurements including macular structure thicknesses were automatically calculated in the 9 ETDRS areas (consisting of a central circular zone with 1-mm diameter, representing the foveal area and inner and outer rings of 3 and 6 mm diameter, respectively) [**14**]. The inner and outer rings were divided into four quadrants: superior, nasal, inferior, and temporal. The mGCC thickness was measured in nine ETDRS subfields (**Fig. 1**). OCT measurement of the eyes of the patients with crNAION was compared to those of the control subjects and unaffected fellow eyes.

**Statistical Analysis**

All numerical data were presented as mean ± standard deviation (SD). Statistical analysis was performed using SPSS 11.5 statistical package program. Categorical variables were compared with the *chi-square* test, while the *Mann Whitney U test* was used for numerical data comparisons between groups. A p-value of less than 0.05 was considered as statistically significant. Spearman correlation analysis was performed to analyze the correlation between VA, mGCC, and pRNFL.

## Results

Patients with crNAION were recruited consecutively at the Dicle University Clinic, School of Medicine, Department of Ophthalmology. A total of 25 patients (16 males and 9 females) with crNAION were eligible for the study. The control group consisted of 50 healthy subjects (26 males and 24 females). The mean age of the patients, and the controls were 58.4±10 years, and 55.9±6.9 years, respectively. There were no statistically significant differences with respect to age and gender between groups (p=0.47, and p=0.50, respectively) (**Table 1**).

**Table 1:** Demographic and clinical characteristics among crNAION

	Chronic (I)	Normal felllow eye (II)	Control (III)	p(I-II)	p(I-III)
Age (year)	58.4±10	N/A	55.9±6.9	N/A	0.47
Gender (M/ F)	16/9	N/A	26/24	N/A	0.5
VA (Snellen)	0.40±0.42	0.8±0.2	1.0	<0.001	<0.001
SE (Diopter)	0.17	0.32	0.39	0.2	0.4
IOP (mmHg)	16.6±4.3	16±3.8	15.7±3.5	0,6	0.4
CMT	254±18	268±24	258±20	0.05	0.4
SE = Spherical equivalent, IOP = Intraocular pressure, VA = Visual acuity, N/ A = not applicable, CMT = Central macular thickness, crNAION = chronic phase of nonarteritic anterior ischemic optic neuropathy					

**Assessment of the pRNFL, mGCC, and CMT in chronic phase of NAION Eyes**

**Fig. 2:**
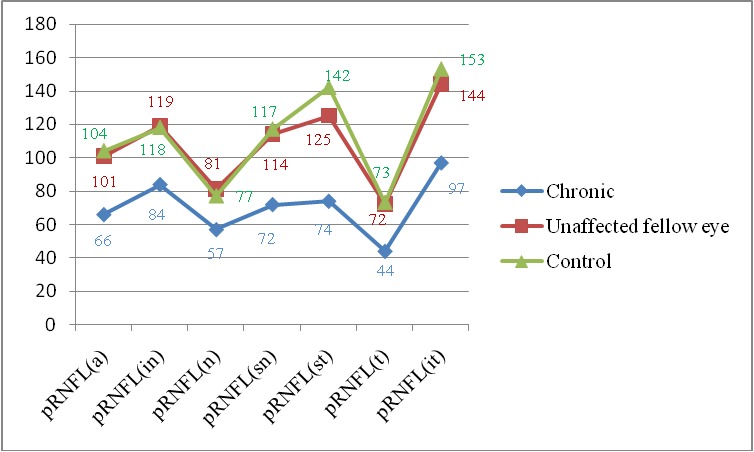
pRNFL in chronic NAION and unaffected fellow eye and control group pRNFL(a) = average, (in) = inferonasal, (sn) = superonasal, (n) = nasal, (st) = superotemporal, (t) = temporal

**Fig. 3:**
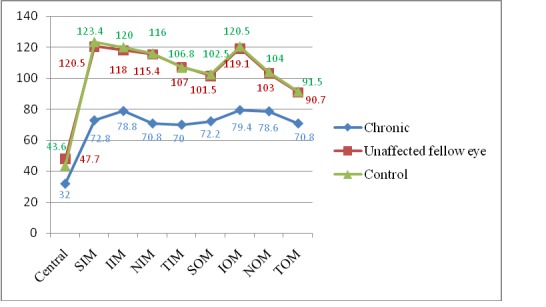
mGCC in chronic phase of NAION and unaffected fellow eye and control group SIM = superior inner quadrant in the macula, NIM = nasal inner quadrant, IIM = inferior inner quadrant, TIM = temporal inner quadrant, SOM = superior outer quadrant in the macula, NOM = nasal outer quadrant, IOM = inferior outer quadrant, TOM = temporal outer quadrant

**Fig. 2** and **3** show the GCC thickness and pRNFL in crNAION eyes. The pRNFL and mGCC thickness in eyes with crNAION were significantly thinner in all quadrants compared to those of healthy controls and unaffected fellow eyes. The CMT of the eyes with chronic phase of NAION was similar to those of control group (**Table 1**).

**Relationship between VA and OCT Measurements**

The VA did not significantly correlate with the GCC thickness and pRNFL thickness in all quadrants of patients with crNAION.

## Discussion

This study showed that mGCC and pRNFL thicknesses were thinner in eyes with crNAION.

RGCs comprise three layers in the retina: the mRNFL (GC axons), the GC layer (GC bodies), and the IPL (GC dendrites). All three layers are defined as the GCC [**6**]. Since the RGCs die as a result of diseases such as NAION, the GCC becomes thinner. Since these layers are directly influenced by GC loss, the GCC scan might be useful in detecting this loss.

Histopathology of NAION involves ischemic edema, cavernous degeneration and atrophy of the axons in the optic nerve as well as apoptosis of RGCs [**5**,**15**,**16**]. It appears that ischemic injury to the axons results in secondary metabolic damage to RGCs culminating in their death. These histopathological changes are difficult to document in vivo. On the other hand, one might be able to indirectly document the loss of RGCs by the measurement of mGCC and the pRNFL, since these ensure quantitative outcomes [**17**–**19**].

Macular parameters obtained by SD-OCT have been investigated in other non-glaucomatous optic neuropathies [**20**]. Segmentation of macular OCT scans enables the in vivo quantification of the integrity of retinal neuronal layers. Furthermore, it analyzes the neuronal integrity since it can demonstrate the thinning of mGCC and pRNFL in chronic optic nerve damage, particularly in NAION [**21**,**22**].

Contreras et al. [**23**] observed that the pRNFL thickness (particularly the superior RNFL thickness) in NAION eyes was increased in the acute phase, becoming thinner thereafter and reaching its lowest level after the 6th month. It has been reported that crNAION causes a decrease in pRNFL thickness [**11**,**23**]. In our study, the pRNFL thickness was decreased in all quadrants of the eyes with crNAION. Our findings were in accordance with literature.

In addition, the association between crNAION and mGCC loss was reported in literature [**6**,**13**]. Our study results demonstrated that the group of patients with crNAION had significantly thinner mGCC thicknesses than the unaffected fellow eye and the control group. We found that the mGCC losses in patients with crNAION were in accordance with literature [**13**,**24**].

However, it is important to remember that changes in OCT layer thickness do not correspond to pathological specificity and that changes may not even occur directly in the layer under study [**25**].

In the present study, a second control group was generated from the unaffected fellow eyes of the NAION patients to eliminate possible confusing factors such as vascular disorders. On the other hand, a relatively small sample size of the study groups was a limitation of this study. The lack of electrophysiological tests or correlation of the visual field with the SD-OCT findings was another limitation. Only Turkish subjects were included in this study, and there may be differences among various ethnicities. Further investigations with a larger sample size including various ethnicities are required to validate our findings.

## Conclusion

We demonstrated that mGCC thickness measured by SD-OCT is capable of detecting axonal damage in crNAION eyes. This study used the SD-OCT and found that mGCC and pRNFL have the ability to detect GC loss in the eyes of patients with crNAION.

**Conflict of Interest**

None 

**Acknowledgments**

The authors have no financial interest in any of the products mentioned in the paper. We are grateful to Dicle University DUBAP for their sponsorship regarding the English editing of this paper.
